# Early anti-VEGF treatment for radiation maculopathy and optic neuropathy: lessons learned

**DOI:** 10.1038/s41433-022-02200-5

**Published:** 2022-08-16

**Authors:** Brittany E. Powell, Kimberly J. Chin, Paul T. Finger

**Affiliations:** 1 The New York Eye Cancer Center, New York, NY USA; 2 Department of Ophthalmology, Fort Belvoir Community Hospital, Fort Belvoir, VA USA

**Keywords:** Radiotherapy, Drug therapy

## Abstract

Radiation therapy has saved both sight and life for eye cancer patients. The most common methods include ophthalmic plaque brachytherapy and external beam techniques. However, subsequent dose-dependent radiation vasculopathy invariably occurs within and around the targeted zone. In 2006, Finger discovered that periodic intravitreal anti-vascular endothelial growth factor (anti-VEGF) bevacizumab could reverse and suppress intraocular radiation vasculopathy. At first, it was administered at the onset of radiation-related vision loss. Though bevacizumab induced regression of macular oedema, retinal haemorrhages and cotton-wool infarcts, most patients were left with residual retinal damage, manifest as metamorphopsia and loss of vision. These results led to earlier and earlier anti-VEGF interventions: first after signs of progressive radiation retinopathy, and then for signs of radiation maculopathy, and finally for high-risk eyes with no clinical signs of retinopathy. Earlier initiation of intravitreal anti-VEGF therapy typically resulted in greater restoration and preservation of macular anatomy, reductions of retinal haemorrhages, resolution of cotton-wool spots and vision preservation. Recent research on optical coherence tomography angiography (OCT-A) has revealed that radiation vasculopathy occurs prior to clinical ophthalmic signs or symptoms. Therefore, it seemed reasonable to consider treating high-risk patients (considered certain to eventually develop radiation maculopathy) to prevent or delay vision loss. Herein, we describe the evolution of treatment for radiation maculopathy as well as recent research supporting anti-VEGF treatment of high-risk patients immediately following radiation to maximize vision outcomes.

## Abstract (zh)

放疗挽救了眼肿瘤患者的视力和生命。最常见的化疗方式包括眼肿瘤近距离治疗和外照射技术。然而, 随后的剂量依赖性辐射性的血管病变总是发生在靶区内和周围。2006年, Finger发现定期玻璃体内注射抗血管内皮生长因子贝伐单抗可以逆转和抑制眼内放射性血管病变。起初, 在与辐射相关的视力丧失开始时服用。虽然贝伐单抗可使黄斑水肿、视网膜出血和棉絮状梗死灶消退, 但大多数患者仍会有视网膜损伤, 表现为视物变形和视力丧失。

这些结果促使尽早使用抗VEGF药物干预: 首先是在出现进行性放射性视网膜病变症状后, 之后是针对放射性黄斑病变症状, 最后是针对没有视网膜病变症状的高危眼。早期开始玻璃体内抗VEGF治疗可更好地恢复黄斑解剖结构, 减少视网膜出血, 治疗棉絮斑并且保护视力。

最近有关光学相干断层扫描血管造影术 (OCT-A) 的研究表明, 放射性血管病变出现于眼科症状或体征之前。因此, 治疗高危患者 (被认为最终会发展为放射性黄斑病变) 以预防或延迟视力丧失似乎是合理的考量。在此, 我们回顾了有关放射性黄斑病变治疗的进展, 以及最近支持放射治疗后立即对高危患者进行抗VEGF治疗以最大限度提高视力的研究。

## Introduction

Radiation has largely replaced enucleation as the treatment of choice for patients with uveal melanoma [1–3]. Overwhelming evidence shows that radiation allows for eye conservation and maintenance of useful vision, and thus improves quality of life [4–6]. However, radiation treatment causes site-specific collateral damage to surrounding structures [7–11]. For example, the most common vision-affecting complications are radiation maculopathy (RM), optic neuropathy (RON) and cataract [12–14]. Of these, RM is the most common cause of severe, irreversible vision loss in eyes treated with radiotherapy for choroidal melanoma [3, 15–19].

### Pathophysiology of radiation vasculopathy

**Fig. 1:**
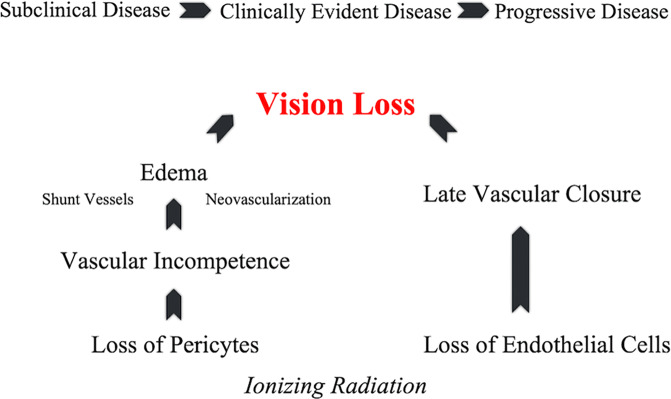
The evolution of radiation retinopathy. Timing and pathophysiologic mechanisms of ionizing radiation-related retinal vasculopathy leading to vision loss.

Radiation therapy results in an occlusive retinal microangiopathy of both the tumour and surrounding vasculature [20–23]. Its impact is related to the radiation dose, dose rate and sensitivity of the exposed tissues. Primarily the by-product of radiation-associated chorioretinal vascular cell damage, RM is characterized by a loss of retinal vascular pericytes and endothelial cells. Pericyte loss drives vascular incompetence seen as microaneurysms and “frosting” (best seen on fluorescein angiography). Secondary leakage of intravascular components (serum, red blood cells, and lipids) presents as oedema, retinal haemorrhage, and exudate. However, radiation-associated loss of endothelial cells results in vascular closure, with downstream ischaemia seen as cotton-wool spots and capillary drop-out [7, 9–11]. These signs of untreated RM are similar to diabetic retinopathy, also characterized by vascular incompetence resulting in early retinal oedema, late retinal ischemia, intraretinal microangiography and neovascularization (Fig. 1).

In both radiation and diabetic retinopathy, anti-VEGF drugs are used to treat a breakdown in the blood-retina barrier which results from the overproduction of VEGF stimulated by ischaemic vasculopathy resulting in vascular permeability, closure, and proliferation. Unsurprisingly, similar treatments have been used for both diabetic retinopathy and radiation retinopathy including: laser photocoagulation, anti-VEGF and corticosteroid medications.

The pathopharmacology of corticosteroids suggests they have anti-VEGF properties, decrease retinal capillary permeability by increasing the activity and density of tight junctions. Steroids decrease the inflammatory effects of radiation, and thus help restore the integrity of the blood-retina barrier [11, 24–26]. Radiation-induced vasculopathy has been described in solid tumours such as lung cancer. There radiation-induced pulmonary vasculopathy mirrors the pathways seen in radiation-induced retinopathy where ionizing radiation leads to vascular compromise, inflammation pulmonary oedema, and pneumonitis which is typically treated with steroids. However no established treatment protocols could be found [24, 27].

### Radiation dose and dose rate effects

**Fig. 2:**
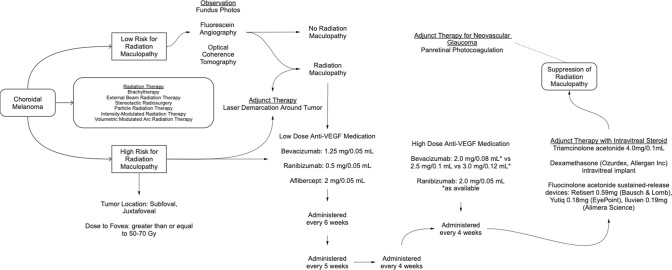
Choroidal melanoma patient care flow chart. This diagram shows our current approach to diagnosis and treatment of radiation maculopathy.

Radiation vasculopathy is radiation dose and dose-rate dependent. The area beneath ophthalmic plaques or target zone receives the highest dose. However, depending upon the plaques’ radiation penumbra, the side-scatter dose of radiation can be significant. In addition, dose gradients (from base to apex) beneath ophthalmic plaque are different, yielding much higher doses when using ruthenium-106 (^106^Ru) versus iodine-125 (^125^I) or palladium-103 (^103^Pd). For example, complete chorioretinal atrophy is commonly seen after ^106^Ru beta-irradiation, not commonly seen after ^103^Pd, and is clinical evidence of relatively high ^106^Ru doses to the tumour’s base. Both larger doses and higher dose-rates can accelerate radiation vasculopathy (Fig. 1) [1, 28–30]. These differences between radiation modalities are reflected in their clinical responses to anti-VEGF treatment for radiation maculopathy and optic neuropathy. In consideration of relative radiation dose and dose-rates to critical ocular structures, successful suppression of radiation maculopathy and optic neuropathy with vision preservation should be more likely after ^103^Pd, ^125^I, proton beam, stereotactic radiosurgery, and ^106^Ru respectively. However, such published evidence is as yet lacking. We performed a PubMed search as outlined in the Methods section and found numerous papers with variable entry criteria and treatment variables leading to the conclusion that no comparative table of results is possible. Therefore, in an effort to better relate our experience with ^103^Pd plaques, we have created a treatment map showing our methods of treatment for radiation maculopathy (Fig. 2).

### Early treatments for intraocular radiation vasculopathy

Prior to the discovery of VEGF and anti-VEGF therapy, there was clinical evidence that retinal ischemia caused radiation retinopathy and neovascular glaucoma [16, 18, 31–34]. Both were found controllable by pan-retinal photocoagulation (PRP) laser or cryodestruction of the ischemic tissues driving intraocular neovascularization. One example is when Finger and Materin discovered that demarcation laser photocoagulation could be used to reduce the circulation within irradiated choroidal melanomas and together with sector PRP (of the radiation-induced hypoxic tissues) prevented or delayed radiation maculopathy [35, 36]. It was only later that studies revealed that choroidal melanoma and ischemic retina were both found to be sources of intraocular VEGF [37, 38]. While ischemic tissue destruction was the only method available to reduce VEGF levels, photocoagulation was less than ideal or not possible when tumours were near, touching, or beneath the fovea and/or optic disc [39]. However, it is still employed for select extramacular and larger tumours with exudative retinal detachments.

### Early intravitreal Anti-VEGF therapy for radiation vasculopathy (2006–2018)

Intravitreal anti-VEGF therapy (IVA) was discovered to provide an exciting new therapeutic option for the treatment of RR, RM and RON [40]. At first, intravitreal anti-VEGF therapy was offered as a treatment to patients considered untreatable with laser photocoagulation and who were actively losing vision due to radiation maculopathy [41, 42]. In those early cases, anti-VEGF treatment was found to result in reductions in cotton-wool spots (CWS), intra-retinal haemorrhages (RH), and macular oedema (MO) with resultant preservation of vision [41, 43]. Heartened by this initial success, over years IVA treatment was subsequently offered to less and less advanced cases. The first such cases included those with metamorphopsia alone and then eyes with signs of CWS, RH or MO without metamorphopsia or vision loss [39, 44, 45]. These cases exhibited clinical signs, optical coherence tomographic (OCT) or fluorescein angiographic (FA) evidence of RM. However, it was noted that despite drug-induced resolution of these clinical and OCT/angiographic findings, at least some evidence of residual retinal destruction persisted [39].

### Current intravitreal Anti-VEGF therapy for radiation vasculopathy

Radiation vasculopathy starts at the time of ophthalmic radiation therapy. Evidence of acute changes can include acute post-treatment oedematous enlargement of the tumour and exacerbation of associated retinal detachments [38]. With time, exudative retinal detachments decline as tumour blood vessels close [46–48]. Optical coherence tomography angiography (OCT-A) research suggests that a subclinical, radiation dose-dependent progressive ischemic vasculopathy continues until retinopathy becomes clinically evident [20–23, 49–51]. Emerging publications on OCT-A imaging have demonstrated microvascular changes occur prior to clinically evident vasculopathy [51, 52]. In addition, optic disc cupping after slotted plaque radiation therapy adds further evidence of post-irradiation progressive microangiopathy [52]. There exist additional, mid-phase signs of radiation-induced occlusive vasculopathy (decreased tumour circulation, reductions in exudative retinal detachments and chorioretinal attenuation) that are often visualized prior to the onset of macular oedema, retinal haemorrhages and cotton-wool spots [48]. For ^103^Pd plaque brachytherapy, these late signs of radiation maculopathy appear at an average of 23.2 months following exposure [7, 20].

Research suggests that prophylactic anti-VEGF therapy can prevent or more likely delay radiation-related retinal damage and loss of vision [39, 53–55]. Herein, we review the available data on early treatment of radiation maculopathy in those patients at the highest risk for developing RM-associated vision loss. Further, we address the risks and potential benefits of intravitreal anti-VEGF treatment prior to the clinical development of RM and RON.

## Methods

A literature search was last conducted in PubMed and the Cochrane Library databases on January 1st, 2022 without date or language restrictions. The search used the following MeSH terms: radiation retinopathy, radiation maculopathy, radiation optic neuropathy, treatment, laser photocoagulation, intraocular, anti-VEGF agents. The search used the following text terms: radiation retinopathy, radiation maculopathy, radiation optic neuropathy, laser photocoagulation, photocoagulation, and intraocular injections.

### Features of radiation maculopathy

#### Medical history

Patients typically present with a history of prior radiation exposure such as plaque brachytherapy (e.g. ^103^Pd,^125^I,^106^Ru) or external beam radiation (e.g. proton, helium ion, stereotactic, photon, or gamma knife radiotherapy). Radiation records should be reviewed for total dose, dose rate and target volume as it relates to normal ocular structures. It is also important to consider the patients' underlying radiation-treatable disease (e.g. ocular adnexal lymphoma, uveal melanoma, metastatic disease, lacrimal gland carcinoma, sinus cancer, and others) [12]. This data can be used to assess the risk of developing radiation vasculopathy and its projected rate of progression. In addition, synchronous systemic disease and medications can affect the incidence and progression of radiation damage [7, 17, 18, 56–58]. Therefore, a careful history and retrieval of medical records offers the potential to maximize patient outcomes.

Eye cancer specialists should also note important surgical information including extraocular plaque/tumour location as it affects both the incidence and location of radiation complications [8, 59]. Radiation dose to fovea and lens has been used to predict RM and cataract after ^103^Pd plaque therapy and thus the risk for secondary, radiation-related vision loss [7, 60–62]. In that tumour and thus plaque location affects dose to critical structures, eyes considered at highest risk for RM after plaque radiation therapy include tumours in subfoveal or juxtafoveal locations in addition to eyes where the dose to fovea was greater than or equal to 50–70 Gy, irrespective of the radiation therapy source [7, 8, 12].

#### Clinical examination

**Fig. 3:**
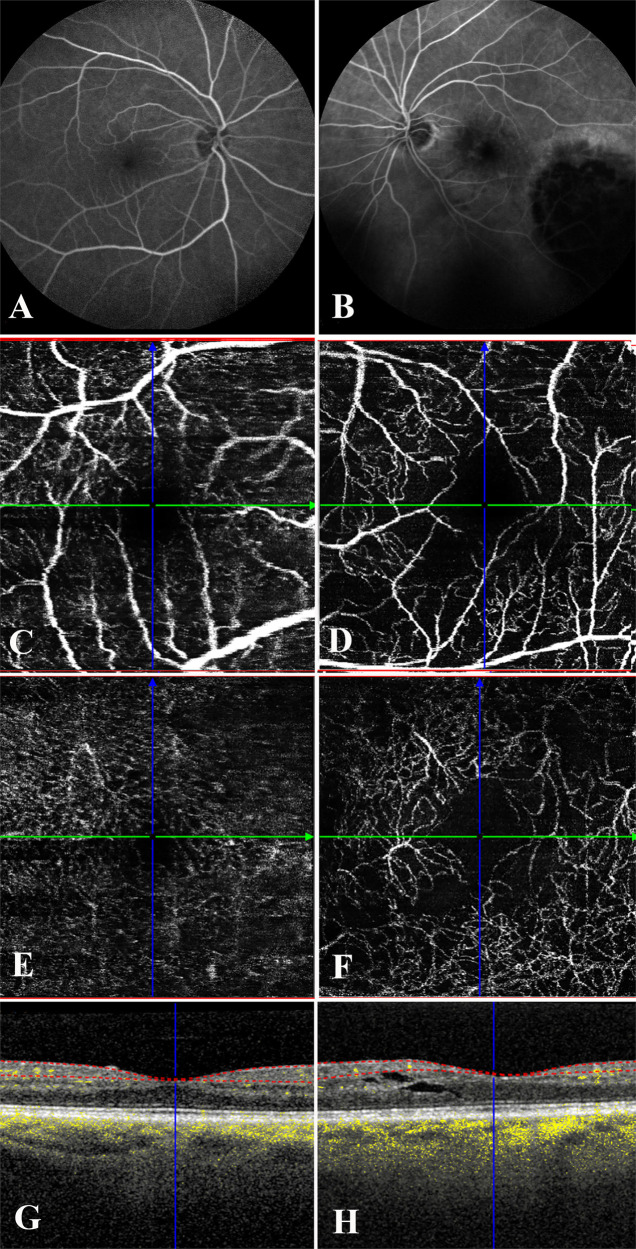
Comparative macular imaging of a normal macula versus the fellow eye with radiation maculopathy. **A** Fluorescein angiography of the right eye is normal right eye. **B** Note perifoveal hyperfluorescence and widening of the foveal avascular zone associated with radiation maculopathy. The tumour is hypofluorescent. **C** Superficial OCT-A of the right normal eye reveals the retinal vessels and capillaries, **D** the left eye OCT-A image reveals capillary drop-out as well as some attenuation of retinal vessels. **E** Deep OCT-A of the right normal eye reveals the deep retinal vessels and capillaries. **F** The left eye OCT-A image reveals capillary drop-out as well as attenuation of deeper retinal vessels. **G** OCT image of the normative right retina is thinner and cohesive as compared to **H**, the irradiated left macular retina with cystoid changes.

**Fig. 4:**
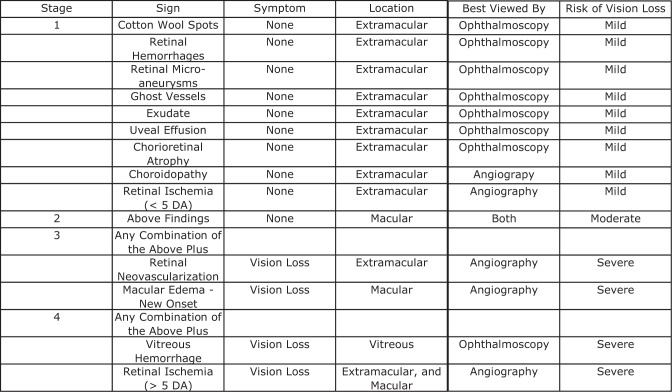
The Finger Staging System for radiation associated vision loss. This table includes signs, symptoms, location, and best method for visualization as related to risk of vision loss with laser or anti-VEGF treatment.

Continuous periodic surveillance is required for the successful care of patients with radiation vasculopathy. For example, with radiation maculopathy the first observable clinical evidence of vascular incompetence is often retinal oedema most easily detectable by comparison of bilateral central foveal thickness measurements on OCT. While fluorescein angiography sometimes reveals macular oedema prior to changes in OCT measurement, OCT and more recently OCT-A play an essential role in both the diagnosis and monitoring of radiation retinopathy (Fig. 3). Shields et al. demonstrated evidence of both superficial and deep capillary plexuses dropout on OCT-A in 65 eyes after plaque radiotherapy of choroidal melanoma suggested these changes could be found in patients without clinical evidence of RM [20]. Sellam et al. observed that these OCT-A features were associated with changes in visual acuity [21, 63, 64]. While these changes appear prior to clinically obvious retinopathy or angiography changes, these OCT-A changes are not well enough understood at this time to modulate treatment. Instead, OCT-A may be used to help determine which patients require initiation of treatment. Currently, anatomic OCT is commonly used to determine macular thickening, as that is usually an early first sign of vascular incompetence related to radiation damage (prior to CWS, retinal haemorrhage, or fluorescein leakage). Progressively later findings include: OCT evidence of intra-retinal cystoid changes, dysmorphic retina, then enlargement of the foveal avascular zone and/or decreased parafoveal capillary density best seen on fluorescein angiography. Early clinical signs of RM may include combinations of CWS, intra-retinal haemorrhages, macular oedema, metamorphopsia, and vision loss. Discovery of clinically apparent RM on ophthalmoscopy or angiography may be complicated by the presence of vitreous haemorrhage and/or tractional retinal detachment [65]. Due to the complexity of secondary findings, Finger devised a staging system that can be used to predict the risk of vision loss associated with ionizing radiation exposure (Fig. 4).

## Results

### Anti-VEGF therapy for anterior radiation optic neuropathy

Radiation optic neuropathy and radiation maculopathy are the leading causes of irreversible vision loss following radiation therapy[1]. While both are characterized by an exudative vasculopathy resulting in oedema and late vascular closure that is dose dependent and the presence of systemic disease and radiation sensitizers, the optic nerve exists within a confined space which results in a more insidious onset [8, 66]. Clinically, RON is characterized by optic disc oedema, haemorrhages, neovascularization, and vision loss [67]. An early form of RON characterized by acute inflammation resulting in optic disc pallor has been reported to occur within several weeks of irradiation, although it can also occur years following treatment [68]. Unsurprisingly given the underlying pathophysiology of RON and the mechanism of action of anti-VEGF agents, they have been shown to preserve vision in those patients with RON [52]. Finger and Chin were the first to evaluate bevacizumab for the treatment of RON in their prospective clinical case series on 14 patients with RON secondary to plaque radiotherapy for choroidal melanoma [69]. Patients were treated with a median of 13 injections of bevacizumab every 6-8 weeks and the results were notable for reductions in the clinical evidence of RON in 100% of the patients (optic disc haemorrhage and oedema) while visual acuity remained stable or improved in 9 of the 14 (64%) patients over a median of 29 months (range 4-39 months). They also noted that 5 (36%) of the patients had clinical evidence of optic atrophy at last follow-up and that 5 (36%) of the patients had a visual acuity of 20/200 or worse at their final follow-up [69]. This study established anti-VEGF as a safe and tolerable treatment for those patients with anterior RON. Other case reports and case series followed with similar results examining intravitreal triamcinolone [70], anti-VEGF [43, 69, 71], and combination treatment [72].

Early treatment of RON has showed conflicting results. Kim et al. showed that patients develop statistically less RON at 24 months with the prophylactic use of bimonthly ranibizumab injections after proton beam therapy [73]; however, this did not result in better visual outcomes [71]. Shah et al. evaluated patients who were prophylactically injected with bevacizumab immediately after plaque removal every 4 months for 2 years and compared them with patients who were treated only with brachytherapy and found no differences in the development of RON, but noted that the actual number of injections administered had a median of 4 injections, which could be an indication that the frequency was insufficient to prevent RON [53]. More recently, Eckstein et al. compared intravitreal therapy with the natural course of RON after primary proton beam therapy for choroidal melanoma and found that patients treated with intravitreal therapy for RON showed no statistically significant differences related to visual acuity or optic atrophy development when compared to those patients who underwent observation only (*p* = 0.579) [74]. This retrospective comparative case series included a total of 93 patients, 48 observed and 43 treated with various intravitreal medications (triamcinolone, bevacizumab, and/or dexamethasone) [74]. One limitation of that study was that all patients receiving intravitreal therapy for RM were excluded from the study resulting in a statistically significant difference between the two groups with regard to tumour proximity to the fovea and macular radiation dose. It is also possible that the observation group had a worse long-term visual outcome because of RM due to a higher macular radiation dose. Lastly, in comparison to the work of Finger and Chin, one must consider that the depth of optic nerve irradiated is longer and the dose-rates higher for proton beam irradiation compared to plaque. While intravenous bevacizumab has been used to suppress intracranial radiation vasculopathy, intravitreal injections of anti-VEGF drugs cannot be expected to reach posterior optic neuropathy [75].

### Anti-VEGF therapy for radiation maculopathy: 10 year data

**Fig. 5:**
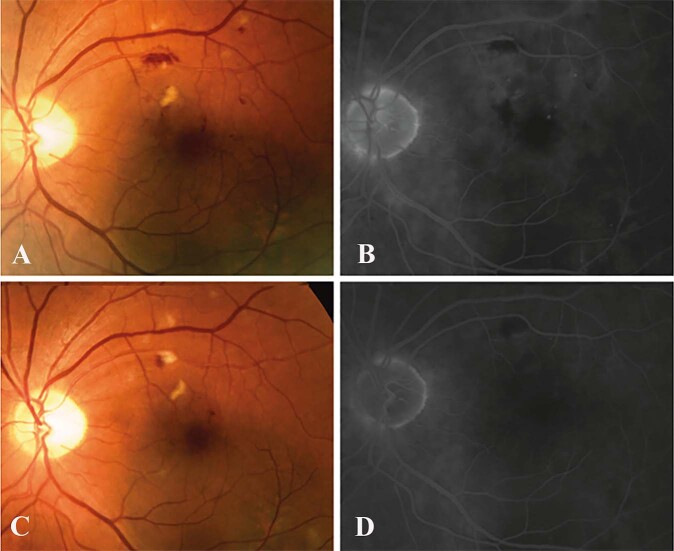
Composite of colour and fluorescein angiographic images over time. **A** Case 1: prior to bevacizumab treatment, the colour photograph reveals retinal haemorrhages, exudates, and intraretinal microangiopathy. **B** The corresponding early fluorescein angiogram reveals macular oedema, capillary nonperfusion, microaneurysms, and focal leakage of neovascular vessels. **C** Three months after treatment with intravitreal bevacizumab, a colour photograph reveals decreased haemorrhages and exudates. **D** The corresponding fluorescein angiogram shows markedly decreased macular oedema, decreased intraretinal microangiopathy, and leakage (sharpening of vessel walls). Published with permission from Anti-Vascular Endothelial Growth Factor Bevacizumab (Avastin) for Radiation Retinopathy, Arch Ophthalmol. 2007;125(6):751-756. Reprinted courtesy of American Medical Association.

Finger et al. were the first to report 10-year data evaluating the benefit of continuous anti-VEGF intravitreal injections for radiation maculopathy [39]. They found that continuous anti-VEGF therapy every 4 to 12 weeks in patients with radiation maculopathy preserved vision: 80% of their 120 patients remained within 2 lines of their initial visual acuity or better with a mean treatment interval of 38 months. The Kaplan–Meier analysis of the probability of remaining within 2 lines of initial visual acuity was 69% at 5 years and 38% at 8 years of anti-VEGF treatment [39]. They also found that continuous, periodic anti-VEGF injections resulted in either a decrease or, more commonly, resolution of the clinical manifestations of radiation maculopathy (Fig. 5). Despite initial clinical improvement and continuous treatment with anti-VEGF injections, most patients developed retinal microaneurysms, capillary nonperfusion, and retinal telangiectasias over time, and a small subset of patients (8%, *n* = 8/99) required adjuvant focal retinal laser photocoagulation of intra-retinal microangiopathy in order to control focal macular oedema and/or retinal neovascularization [39]. Therefore, even suppressed radiation maculopathy was progressive. However, this work established anti-VEGF therapy as a well-tolerated, safe, and effective means to preserve vision in those with radiation maculopathy [39]. This long-term study also revealed that, despite initial adequate suppression, patients required more intensive treatment over time: shorter intervals between medical doses, increased amounts of medication, switching to other medications, and eventual added steroid polypharmacy [39, 76]. This data was consistent with that seen with other chronic progressive diseases (e.g., diabetes, hypertension, heart disease) where progressively earlier intervention offers better outcomes, which sets the stage for additional research.

### The prevent or delay study

#### Defining the high-risk group

Prior research has shown that there exists a subset of patients at exceptionally high risk of developing RM. For example, in a prior study of 67 eyes that received a ^103^Pd dose to fovea greater than or equal to 70 Gy showed a mean visual acuity near 20/40 prior to radiation, which dropped to 20/800 at last follow-up (a mean of 47.3 months) [7]. In another ^103^Pd study, patients with subfoveal melanomas treated to a mean fovea dose of 157.7 Gy and mean pre-treatment visual acuity of 20/50 decreased to a median final visual acuity of 20/180 (at 62.2 months, 16 eyes treated for radiation maculopathy) [28]. Thus, patients with subfoveal melanomas carry the highest risk for radiation maculopathy and vision loss due to tumour location associated high radiation doses, plus they were not eligible for laser photocoagulation-induced VEGF suppression.

**Fig. 6:**
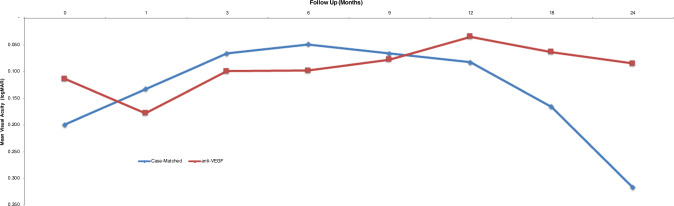
Mean visual acuity (Log MAR, converted from ETDRS). The line graph depicts all patients in the anti-VEGF group receiving early intervention with intravitreal injections versus the patients in the case-matched group over time. ETDRS Early Treatment Diabetic Retinopathy Study. Original figure from Anti-VEGF Therapy Immediately after Plaque Radiation Prevents of Delays Radiation Maculopathy, *Ophthalmology Retina*, 2020 May;4(5):547–50, reprinted courtesy of Elsevier Science Publishers.

In consideration that tumour location and dose influence visual acuity outcomes, Powell and Finger performed a retrospective, case-matched study to determine the efficacy of continuous intravitreal anti-VEGF therapy for patients at high-risk for vision loss [51]. Fourteen patients were diagnosed with choroidal melanoma, treated with ^103^Pd plaque radiotherapy, and then treated with monthly anti-VEGF injections. Periodic intravitreal anti-VEGF therapy was initiated prior to the onset of RM at a mean of 24 days of plaque placement [55]. These patients were case-matched—by radiation dose to fovea, proximity to fovea, and size of tumour to 14 patients similarly diagnosed and treated with radiation therapy only for choroidal melanoma between 1999-2005 (prior to the advent of anti-VEGF therapy) [40]. The mean fovea dose for the anti-VEGF treated and case-matched groups were similar, at 108.0 and 108.4 Grey *(p* = 0.981), respectively. Therefore, both groups studied were at highest risk of developing RM due to high foveal ^103^Pd dose. The mean visual acuity between the two groups was not statistically significantly different at the time of diagnosis. However, the visual acuities differed significantly at last follow-up. The anti-VEGF group showed an overall last mean visual acuity of 20/32, as compared with the case-matched group whose last mean visual acuity was 20/160. Five of the anti-VEGF patients versus 2 case-matched eyes showed improvement in visual acuity. When compared with their initial visual acuities, 9 patients (64.3%) in the anti-VEGF treated group showed improvement or no change in visual acuity, as compared to only 4 patients (28.6%) in the case-matched group *(p* = 0.054). At the last follow-up examination, 5 patients (35.7%) in the anti-VEGF group were within 2 lines of the pre-treatment visual acuity versus 0 of the patients in the case-matched group *(p* = 0.020). None of the patients in the anti-VEGF group lost more than 3 lines of vision, compared with 10 patients (71.4%) in the case-matched group *(p* = *<*0.001). Improvements in visual acuity were typically due to resolution of the exudative retinal detachments and intra-retinal fluid. Initial visual acuities between the groups remained similar until 9 months after treatment. Then, the anti-VEGF group remained stable, whereas the case-matched group’s vision significantly declined (Fig. 6) [51].

#### OCT imaging on the anti-VEGF treated group

At a mean 32 months after irradiation, the anti-VEGF treatment group experienced a mean decrease of 172 microns of central foveal thickness (CFT) compared to their pre-treatment measurements. Macular anatomy evaluations on the bevacizumab group suggested that maintaining CFT resulted was associated with preservation of vision. The absence of complications related to continuous, periodic, intravitreal bevacizumab therapy (up to 53 months follow up), were consistent with our prior studies [39, 41, 42]. Radiation retinopathy was graded using the Finger staging system for radiation retinopathy (Fig. 4). At last follow-up, 7 anti-VEGF patients (50%) demonstrated RM, as compared with 12 patients (85.7%) in the case-matched group. There were no patients in either group with Stage 1 radiation retinopathy. 42.9% (*n* = 6) of patients in the bevacizumab group had developed Stage 2 radiation retinopathy, versus 7.1% (*n* = 1) of the control group *(p* = 0.036). Also significant was the 7.1% (*n* = 1) of patients in the bevacizumab group with Stage 3 radiation retinopathy, compared to 64.3% (*n* = 9) of the patients in the control group *(p* = 0.002). Not noted to be of statistical significance was the comparison of patients that developed Stage 4 radiation retinopathy: none of the patients in the bevacizumab group developed Stage 4 versus 14.3% (*n* = 2) of the control group. Of the anti-VEGF patients, 6 showed solitary cotton-wool spots, 1 with a single intra-retinal cyst (evident on OCT) that subsequently resolved with continued anti-VEGF injections. The 7 patients who did not develop RM had a mean of 33 months of follow-up. In summary, patients who received periodic, continuous intravitreal bevacizumab starting within 6 months of plaque irradiation were less likely to develop clinically significant radiation retinopathy.

## Discussion

Herein, we have presented our initial clinical experience, subsequent evolution and most recent investigation of the use of intravitreal anti-VEGF therapy to treat radiation maculopathy. While intravitreal anti-VEGF drugs offer a potent treatment, it is a time-limited suppressive effect. Modulation of anti-VEGF drug, drug dose, time-intervals (progressively shorter) and late polypharmacy with steroids are required to maintain patient vision. In addition, our most recent study suggests that early treatment offers the best chance to prevent or (more likely) delay radiation vasculopathy associated loss of vision.

These results are consistent with other studies using prophylactic anti-VEGF bevacizumab for patients undergoing plaque radiotherapy [53, 54]. Shah et al. treated patients with bevacizumab every 4 months for 2 years starting at the time of plaque removal and compared their outcomes to a control group of patients who opted out of treatment with bevacizumab [53]. They reported that the proportion of patients with moderate vision loss of three or more lines in the study was 33% (*n* = 96/292) in the bevacizumab group versus 57% (*n* = 72/126) for the controls (*p* < 0.001). In addition, they found the cumulative incidence of OCT-evident macular oedema over 2 years to be 26% in the bevacizumab group versus 40% for the control eyes (*p* = 0.004). Similarly, clinical evidence of RM was noted in 16% in the bevacizumab group versus 31% in the controls (*p* = 0.001). However, it is important to note that this study utilized longer than normative (4-month) intervals between anti-VEGF doses. Shields et al. also looked at their 4-year data from 1,131 eyes that had were treated with intravitreal bevacizumab at that longer than normative 4-month intervals for 2 years, and recorded the visual outcome was better at 1, 2, 3, and 4 years. Their 4-year median visual acuity of 20/70 was compared to counting fingers in their non-randomized, control (i.e. non-bevacizumab) group [54]. One large difference between our study and the work done by Shah and Shields is that their studies did not differentiate between those choroidal melanomas at highest risk of developing radiation complications based on tumour location or radiation dose to fovea. Despite these differences, their data strongly supports the use of prophylactic intravitreal anti-VEGF treatment to preserve vision after ophthalmic plaque radiation therapy.

Currently, the authors of this paper typically initiate prophylactic intravitreal anti-VEGF treatment for patients based on calculated radiation dose to fovea or optic nerve and start at the time of plaque removal. Then every six weeks, followed by decreases in the frequency of treatment and/or increased dose of anti-VEGF treatment, based on serial examinations, retinal photography and OCT. Thus, multimodality retinal imaging is used to titrate treatment rather than as marker to initiate treatment.

Additional support for early anti-VEGF treatment of eyes at risk for radiation-associated vision loss can be found in recent OCT-A research. This modality has been shown to detect subclinical radiation vasculopathy, thus enabling early detection [21, 49, 50, 52, 77, 78]. However, we also acknowledge that those OCT-A findings are not well enough understood to be used to modulate treatment. Future research could focus on better understanding the pathophysiology of ophthalmic radiation vasculopathy. Then, perhaps, OCT-A findings could be used to determine which patients require intervention and to modulate how often an intervention is needed to prevent or delay vision-threatening radiation oculopathy and thus prevent or delay radiation-associated loss of vision.

### Summary

Intravitreal anti-VEGF has proven to be a well-tolerated method to suppress radiation maculopathy and thereby preserve vision. Early intervention can be used to suppress clinical manifestation of vision-threatening radiation maculopathy in high-risk patients. However, the differences between radiation modalities that affect the efficacy of intravitreal anti-VEGF therapy must also be considered.

### Disclaimer

The views expressed in this submission reflect the results of research conducted by the authors and do not necessarily reflect the official policy or position of the Department of the Navy, Department of Defense, or the U.S. Government.
